# Supplementary material for: Impact of the COVID-19 pandemic on human-nature relations in a remote nature-based tourism destination
Source: PLoS One. 2022 Sep 29;17(9):e0273354. doi: 10.1371/journal.pone.0273354 (PMC9521831; doi:10.1371/journal.pone.0273354)
Supplement: Attachment — Submitted filename: PONE-D-22-04631_reviewer2.pdf [file pone.0273354.s001.pdf]

PLOS ONE

Impact of the COVID-19 pandemic on human-nature relations in a remote nature-based tourism destination

--Manuscript Draft--

**Manuscript Number:**	PONE-D-22-04631
**Article Type:**	Research Article
**Full Title:**	Impact of the COVID-19 pandemic on human-nature relations in a remote nature-based tourism destination
**Short Title:**	Impact of the COVID-19 pandemic on human-nature relations in a remote nature-based tourism destination
**Corresponding Author:**	Evert Mul, Ph.D.<br>NINA: Norsk Institutt for Naturforskning<br>9007 Tromsø, NORWAY
**Keywords:**	Nature-based recreation; COVID-19; northern Norway; tourism; Flickr; structural topic model
**Abstract:**	Tourism and nature-based recreation has changed dramatically during the COVID-19 pandemic. Travel restrictions caused sharp declines in visitation numbers, particularly in remote areas, such as northern Norway. In addition, the pandemic may have altered visitor behaviour and preferences. We studied visitor numbers and behaviour in northern Norway, based on user-generated data, in the form of photographic material that was uploaded to the popular online platform Flickr. Photographs were subjected to the “Cloud Vision” automatic content analysis algorithm from Google. The resulting collection of labels that were assigned to each photograph was analysed in structural topic models, using photography date (relative to the start of the pandemic) and reported or estimated photographers nationality as explanatory variables. Our results show that nature-based recreation relating to “mountains” and “winter” became more prevalent during the pandemic, amongst both domestic and international photographers. Shifts in preferences due to the pandemic outbreak strongly depended on nationality, with domestic visitors demonstrating a wide interest in topics (including urban, cultural, and nature-based recreation) while international visitors maintained their preference for nature-based experiences. Among those activities that suffered the most from decline in international tourism was northern lights and cruises as indicated by the topic models. On the other hand, images depicting mountains and flora and fauna increased their prevalence during the pandemic. Domestic visitors, on the other hand, spent more time in urban settings as a result of restrictions, which results in a higher prevalence of non-nature related images. This contrast in flexibility to adapt to changing conditions and travel restrictions should be incorporated in collaborative efforts of municipalities and tour operators to develop sustainable local nature-based tourism products, particularly in remote areas.
**Order of Authors:**	Evert Mul<br>Francisco Javier Ancin Murguzur<br>Vera Helene Hausner
**Additional Information:**	
**Question**	**Response**
**Financial Disclosure**<br><br>Enter a financial disclosure statement that describes the sources of funding for the work included in this submission. Review the submission guidelines for detailed requirements. View published research articles from *PLOS ONE* for specific	This study was financed by the Norwegian Research Council project BlueTrans- “Ocean Health under Blue Transitions”, project no. 280778<br><br>The publication charges for this article have been funded by a grant from the publication fund of UiT The Arctic University of Norway.<br><br>The funders had no role in study design, data collection and analysis, decision to publish, or preparation of the manuscript.

Powered by Editorial Manager® and ProduXion Manager® from Aries Systems Corporation

examples.<br><br>This statement is required for submission and **will appear in the published article** if the submission is accepted. Please make sure it is accurate.<br><br>**Unfunded studies**<br>Enter: *The author(s) received no specific funding for this work.*<br><br>**Funded studies**<br>Enter a statement with the following details:<br>• Initials of the authors who received each award<br>• Grant numbers awarded to each author<br>• The full name of each funder<br>• URL of each funder website<br>• Did the sponsors or funders play any role in the study design, data collection and analysis, decision to publish, or preparation of the manuscript?<br>• **NO** - Include this sentence at the end of your statement: *The funders had no role in study design, data collection and analysis, decision to publish, or preparation of the manuscript.*<br>• **YES** - Specify the role(s) played.<br><br>* typeset	
**Competing Interests**<br><br>Use the instructions below to enter a competing interest statement for this submission. On behalf of all authors, disclose any competing interests that could be perceived to bias this work—acknowledging all financial support and any other relevant financial or non-financial competing interests.<br><br>This statement is required for submission and **will appear in the published article** if the submission is accepted. Please make sure it is accurate and that any funding sources listed in your Funding Information later in the submission form are also declared in your Financial Disclosure statement.	The authors have declared that no competing interests exist.

Powered by Editorial Manager® and ProduXion Manager® from Aries Systems Corporation

View published research articles from *PLOS ONE* for specific examples.<br><br>**NO authors have competing interests**<br><br>Enter: *The authors have declared that no competing interests exist.*<br><br>**Authors with competing interests**<br><br>Enter competing interest details beginning with this statement:<br><br>*I have read the journal's policy and the authors of this manuscript have the following competing interests: [insert competing interests here]*<br><br>* typeset	
**Ethics Statement**<br><br>Enter an ethics statement for this submission. This statement is required if the study involved:<br><br>• Human participants<br>• Human specimens or tissue<br>• Vertebrate animals or cephalopods<br>• Vertebrate embryos or tissues<br>• Field research<br><br>Write "N/A" if the submission does not require an ethics statement.<br><br>General guidance is provided below. Consult the submission guidelines for detailed instructions. **Make sure that all information entered here is included in the Methods section of the manuscript.**	N/A

**Format for specific study types**<br><br>**Human Subject Research (involving human participants and/or tissue)**<br>• Give the name of the institutional review board or ethics committee that approved the study<br>• Include the approval number and/or a statement indicating approval of this research<br>• Indicate the form of consent obtained (written/oral) or the reason that consent was not obtained (e.g. the data were analyzed anonymously)<br><br>**Animal Research (involving vertebrate animals, embryos or tissues)**<br>• Provide the name of the Institutional Animal Care and Use Committee (IACUC) or other relevant ethics board that reviewed the study protocol, and indicate whether they approved this research or granted a formal waiver of ethical approval<br>• Include an approval number if one was obtained<br>• If the study involved *non-human primates*, add *additional details* about animal welfare and steps taken to ameliorate suffering<br>• If anesthesia, euthanasia, or any kind of animal sacrifice is part of the study, include briefly which substances and/or methods were applied<br><br>**Field Research**<br><br>Include the following details if this study involves the collection of plant, animal, or other materials from a natural setting:<br>• Field permit number<br>• Name of the institution or relevant body that granted permission	
**Data Availability**<br><br>Authors are required to make all data underlying the findings described fully available, without restriction, and from the time of publication. PLOS allows rare exceptions to address legal and ethical concerns. See the PLOS Data Policy and FAQ for detailed information.	Yes - all data are fully available without restriction

Powered by Editorial Manager® and ProduXion Manager® from Aries Systems Corporation

A Data Availability Statement describing where the data can be found is required at submission. Your answers to this question constitute the Data Availability Statement and **will be published in the article**, if accepted.<br><br>**Important:** Stating 'data available on request from the author' is not sufficient. If your data are only available upon request, select 'No' for the first question and explain your exceptional situation in the text box.<br><br>Do the authors confirm that all data underlying the findings described in their manuscript are fully available without restriction?	
**Describe where the data may be found in full sentences. If you are copying our sample text, replace any instances of XXX with the appropriate details.**<br><br>• If the data are **held or will be held in a public repository**, include URLs, accession numbers or DOIs. If this information will only be available after acceptance, indicate this by ticking the box below. For example: *All XXX files are available from the XXX database (accession number(s) XXX, XXX.).*<br>• If the data are all contained **within the manuscript and/or Supporting Information files**, enter the following: *All relevant data are within the manuscript and its Supporting Information files.*<br>• If neither of these applies but you are able to provide **details of access elsewhere**, with or without limitations, please do so. For example:<br><br>*Data cannot be shared publicly because of [XXX]. Data are available from the XXX Institutional Data Access / Ethics Committee (contact via XXX) for researchers who meet the criteria for access to confidential data.*<br><br>*The data underlying the results presented in the study are available from (include the name of the third party*	All data files will be available on the "opendata.uit.no" database from the university of Tromsø, after acceptence.

Powered by Editorial Manager® and ProduXion Manager® from Aries Systems Corporation

and contact information or URL).<br>• This text is appropriate if the data are owned by a third party and authors do not have permission to share the data.<br><br>* typeset	
Additional data availability information:	Tick here if the URLs/accession numbers/DOIs will be available only after acceptance of the manuscript for publication so that we can ensure their inclusion before publication.

Powered by Editorial Manager® and ProduXion Manager® from Aries Systems Corporation

Manuscript
Click here to access/download;Manuscript;MANUSCRIPT.docx

Impact of the COVID-19 pandemic on human-nature relations in a remote nature-based tourism destination

Evert Mul[1, *], Francisco Javier Ancin Murguzur[1], Vera Helene Hausner[1]

[1] Institute of Arctic and Marine Biology, UiT – The Arctic University of Norway, NO-9019 Tromsø, Norway

[*] Current address: Norwegian Institute for Nature Research (NINA), Fram Centre, NO-9296 Tromsø, Norway

Abstract

Tourism and nature-based recreation has changed dramatically during the COVID-19 pandemic. Travel restrictions caused sharp declines in visitation numbers, particularly in remote areas, such as northern Norway. In addition, the pandemic may have altered visitor behaviour and preferences. We studied visitor numbers and behaviour in northern Norway, based on user-generated data, in the form of photographic material that was uploaded to the popular online platform Flickr. Photographs were subjected to the “Cloud Vision” automatic content analysis algorithm from Google. The resulting collection of labels that were assigned to each photograph was analysed in structural topic models, using photography date (relative to the start of the pandemic) and reported or estimated photographers nationality as explanatory variables. Our results show that nature-based recreation relating to “mountains” and “winter” became more prevalent during the

pandemic, amongst both domestic and international photographers. Shifts in preferences due to the pandemic outbreak strongly depended on nationality, with domestic visitors demonstrating a wide interest in topics (including urban, cultural, and nature-based recreation) while international visitors maintained their preference for nature-based experiences. Among those activities that suffered the most from decline in international tourism was northern lights and cruises as indicated by the topic models. On the other hand, images depicting mountains and flora and fauna increased their prevalence during the pandemic. Domestic visitors, on the other hand, spent more time in urban settings as a result of restrictions, which results in a higher prevalence of non-nature related images. This contrast in flexibility to adapt to changing conditions and travel restrictions should be incorporated in collaborative efforts of municipalities and tour operators to develop sustainable local nature-based tourism products, particularly in remote areas.

Keywords

Nature-based recreation, COVID-19, northern Norway, tourism, Flickr, structural topic model

Introduction

The global responses to the COVID-19 pandemic have had wide-reaching impacts on nature-based recreation and tourism (OECD, 2020; Sigala, 2020) and on human-nature interactions in general (Soga et al., 2021). Since the beginning of the pandemic in 2020, protected area visitation and wildlife tourism has decreased all over the world (Spenceley et al., 2021). On average, the global number of international tourist arrivals in the world in 2020 was 73%

lower compared to 2019. Between April and December 2020, the average reduction was more than 85%, compared to the same months in 2019 (Calculated from: World Tourism Organization United Nations, 2021). This decrease in visitations has a wide variety of consequences for both nature and society, ranging from job-loss (Sigala, 2020), to fewer vehicle-wildlife collisions (Shilling et al., 2021), behavioural changes in wildlife (Rutz et al., 2020), increased opportunity for poaching (Spalding et al., 2020) and a reduction in crowd-sourced scientific data, such as wildlife observations (Rose et al., 2020).

However, some nature areas experienced increasing numbers of visitors during the pandemic. Empirical data shows an increased number of visitations in areas that are easily accessible from urban areas, such as green spaces in cities (Morse et al., 2020; Venter et al., 2020), or forests that are with short distance from urban areas (Derks et al., 2020). As a result of the pandemic, nature-recreationists opted to focus on nearby natural areas, as opposed to remote areas. For example, recreational bird-watchers increasingly visited nature areas closer to home (Randler et al., 2020). This emphasizes the need to consider area-accessibility when evaluating effects of COVID-19 responses on nature-based tourism. Remote areas might experience different, or even antagonistic consequences, compared to areas that are easily accessible. To reduce the spread of COVID-19, many governments imposed local and international travel restrictions. As a result, remote areas throughout the world experienced drastic reductions in visitations (Spenceley et al., 2021).

Measuring the consequences of changes in visitation rate, or spatial and temporal recreational use in remote destinations can be challenging. Some studies have relied on user-surveys to evaluate trends in recreational activities (Morse et al., 2020; Randler et al., 2020), whereas another avenue is to explore “big data” to empirically examine change in

tourism and outdoor recreation patterns (Jaung, 2021; Rice & Pan, 2021). In general, three forms of "Big data" can be identified: transaction data, device data, and user-generated data (Li et al., 2018). Transaction data refers to data that is generated from web searches, (online) bookings, or consumer cards. Device data includes data that is generated by mobile phones and other electronic devices, which for example provide information about the owners location through for GPS, Bluetooth and WIFI (Shoval & Ahas, 2016). Finally, user-generated data consists of information that is shared online by the users, such as through blogs, online reviews, or images that are posted on photo-sharing websites (Li et al., 2018). Rice & Pan (2021) used an open-source Google dataset, based on aggregated location data from smartphones (device data), in order to determine changes in park visitations. Peng et al. (2017) used web search data (transaction data) to predict the volume of tourism visitations in the Jiuzhai Valley (China). Other studies have employed data from social media platforms such as Twitter, Instagram and Flickr (user-generated data) to identify spatiotemporal trends in nature-based recreation at global and regional scales (Mancini et al., 2018; Runge et al., 2020; Wood et al., 2020). Flickr is particularly suitable for understanding spatial behaviour of nature-based tourism and recreation, as those using this platform are highly engaged in nature (Muñoz et al., 2020; Toivonen et al., 2019). Data from this platform has been shown to correspond to on-site visitor data on tourism and recreation. This type of data allows for automatic content analysis and provides opportunities for linking recreational use to e.g. country of origin (Richards et al., 2018; Runge et al., 2020; Wood et al., 2020).

In a user survey of 3.204 residents in Vermont in USA, Morse et al. (2020) demonstrated that the spatial shifts and the non-material contribution of nature differs with respect to type of activities and socio-demographics. For instance, camping and social recreational activities, climbing and to some extent boating declined during the pandemic, whereas other activities

such as gardening, hiking, jogging, and nature photography increased. There was also a significant difference between urban and rural areas with respect to the recreational activities that people engaged in. Their results conform with previous studies that have found rural residents, domestic – and international visitors to differ in their spatial use of landscapes and the cultural ecosystem services they prefer (Ghermandi et al., 2020; Muñoz et al., 2019). The difference between domestic and international visitors is particularly important for nature-based tourism destinations that rely upon domestic visitors to mitigate the decline of international visitors. Spenceley et al. (2021) suggest that tourism destinations need to draw on some of the lessons from the COVID-19 pandemic and build resilience by offering activities and products that appeal to both domestic and international tourists.

In this study, we use automatic content analysis of pictures that were uploaded to the Flickr platform, combined with structured topic modelling, to examine the shift in spatial distribution of nature-based tourism and recreation in northern Norway, a remote nature-based tourism destination. This region experienced a boom in national and international visitation prior to the COVID-19 pandemic (Innovasjon Norge, 2019). However, in 2020, the international tourist arrivals in Norway decreased by 77%, compared to 2019, which is noticeably stronger than the European average decrease in visitations (-70%) or the global average (-73%) (World Tourism Organization United Nations, 2021). When considering only arrivals after March 2020 (when the pandemic reached Norway), the decrease is more than 80%, compared to 2019. We investigate how appreciation of nature has shifted before and during the COVID-19 and explore the differences between domestic and international visitors. More specifically, we formulated four research questions:

1) How are visitation numbers influenced by the COVID-19 pandemic, based on photographers that were active on the Flickr platform?
2) What were the most popular photography topics in Northern Norway?
3) How did the pandemic affect the prevalence of these photographic topics amongst international and domestic photographers separately?
4) How did the prevalence of photography topics differ between international and domestic photographers, and was the compared prevalence influenced by the pandemic?

Methods

Data collection

The data that was used in this paper consists of part of the dataset that was compiled by Runge et al. (2020), complemented by additional, more recent data. This dataset is based on photographs that users uploaded to the social media platform Flickr (www.flickr.com). Photos that were taken between 2001 and April 2021 in Northern Norway and which were uploaded to Flickr were processed using an automated content-identification algorithm: Google's Cloud Vision. The initial dataset was extracted from Flickr on 4 December 2017 (Runge et al., 2020), while additional data was extracted between November 2020 and June 2021. Only photos that were geotagged (assigned to a location) were included in the dataset. Since this study is focused on northern Norway, we only extracted photos that were taken north of 65°N, and between 0° and 35°E. Finally, we removed the remaining photos that were taken in neighbouring countries (Finland & Sweden). We used "R" statistical computing software (version 4.0.3) for all data processing, filtering and further analysis (R

Core Team, 2020). Google's Cloud Vision algorithm was used to assign labels to photos to describe its content using the "Rooglevision" package in R (Teschner, 2021).

For each photograph, we stored only the data and location of each photograph as well as a number representing each photographer. In addition, we stored up to 20 labels that were assigned to each photo by the Cloud Vision algorithm: we set the confidence threshold of the labelling algorithm to 50% to ensure an accurate description of the image contents. For each photographer, the country of origin was either extracted from the information the photographers shared on the Flickr platform, or, if no country of origin was shared, it was estimated based on the locations of the photographer's photos. For that purpose, we calculated the arithmetic mean of all geotagged photographs taken by the visitors.

As an initial exploration of the effects of the measures to prevent the spread of Covid-19, we studied the annual number of photographers, and the annual ratio between international and Norwegian photographers. Furthermore, we compared the percentage of Norwegian photographers before and after the implementation of these measures.

Structural topic model

We used a Structural Topic Model to identify topics from the labels that have been assigned to each photograph (Roberts et al., 2014). A Structural Topic Model is a machine learning approach to identify unobserved groups of labels, or latent topics (Lucas et al., 2015). In principle, STMs estimate how associated each word is to each topic. At the same time, it estimates to which extent each picture is associated to each topic. Words can be associated to multiple topics, and pictures can be associated to multiple topics. The number of latent topics needs to be determined by the user beforehand. It is possible to compare statistical properties of models with a different number of topics to select the best model. However,

since the purpose of these models is to evaluate the semantic coherence of a collection of texts, the strongest statistical models may not necessarily represent the best classification of latent topics (e.g. Grimmer & Stewart, 2013). Instead, we opted to manually compare a range of models with a varying number of topics. Based on the most prevalent words within each topic, we arbitrary selected the model that contained the most coherent and distinguishable selection of topics, within the context of this study. Following this procedure, we selected a model with 8 topics. All spaces, punctuations, and special characters within each label were removed, to create a collection of singe-word "terms", which can be analysed by a STM. To improve the fit of the model, labels that occurred less than 10 times, or more than 90.000 times in the entire dataset were removed. The upper limit was selected to remove a single label: "sky" which was allocated to almost half of the photos by the Cloud Vision algorithm. As a result, this label has relatively little value in the identification of latent topics, as it fits in all topics. STMs were fitted using the "STM" package in R (Roberts et al., 2019).

Regression model

STM's differ from other topic models, as they can incorporate metadata to evaluate the influence of covariates in a regression-type analysis (Roberts et al., 2019). This enabled us to incorporate background information about the photographer (domestic or international) and information about when the photograph was taken (before or after the COVID-19 measures were implemented). As the covariate model structure influences the topic modelling procedure, there is no method to compare the statistical quality of models with different structures, as is common in other statistical models (e.g. by comparing the estimated prediction error, such as the AIC estimator). However, STMs can be used to study

the effect of covariates on the distribution of words (labels) amongst the topics, based on a user-defined covariate model structure. Here we studied the interacting effect of two covariates: whether the picture was taken before or after implementation of corona measures, and whether the photographer was Norwegian or international.

Results

Dataset and photographer country of origin

The dataset contains information about 195.200 photos that were taken in northern Norway by 5.247 photographers between 01-01-2000 and 31-03-2021 and uploaded to the Flickr website. Note that the number of photos currently available on Flickr might differ, as users can remove or add pictures. Interannual variability in the number of photographs and photographers indicated an increase in the use of the Flickr platform that peaked around 2013/2014 (Table 1). After 2014, the use of Flickr decreased steadily, as indicated by the decrease in number of photographers. However, in 2020, this decline was amplified drastically, as the number of photographers decreased from 429 in 2019 to 205 in 2020, which is the lowest number of photographers since 2006. Since only the first three months of 2021 were included in the dataset, no comparison between 2020 and 2021 was made (Table 1).

*Table 1 – Annual number of international and Norwegian photographers. * The dataset only includes the first three months of 2021.*

NUMBER OF PHOTOGRAPHERS

YEAR	International	Norway	**Total**	**%Change**
2000	8	4	**12**	
2001	7	3	**10**	**-17%**
2002	12	6	**18**	**80%**
2003	16	6	**22**	**22%**
2004	30	12	**42**	**91%**
2005	36	16	**52**	**24%**
2006	102	38	**140**	**169%**

2007	156	71	**227**	**62%**
2008	231	89	**320**	**41%**
2009	276	109	**385**	**20%**
2010	385	143	**528**	**37%**
2011	550	170	**720**	**36%**
2012	561	172	**733**	**2%**
2013	644	157	**801**	**9%**
2014	651	129	**780**	**-3%**
2015	617	136	**753**	**-3%**
2016	610	119	**729**	**-3%**
2017	543	104	**647**	**-11%**
2018	422	54	**476**	**-26%**
2019	383	46	**429**	**-10%**
2020	176	38	**214**	**-50%**
2021*	29	16	**45**	**NA**

The country of origin was known for 1.731 of the photographers (93.998 photos) and estimated for the remaining 3.516 photographers (101.202 photos). According to the downloaded and estimated photographer origins, the data consisted of 565 Norwegian and 4.682 non-Norwegian photographers. Overall, Norwegian photographers took approximately 16 % of the photos (31.663). From 2019 to 2020, the number of international photographers dropped by 54%, while the number of Norwegian photographers dropped by only 17% in the same year. However, we have no explanation for the sharp reduction of 48% in the number of Norwegian photographers in the year 2018 (Table 1). We marked 1 April 2020 as the implementation date for the measures to prevent the spread of Covid-19 in Norway. Before that date, 11% of the photographers were Norwegian, while that percentage increased to 26% after the implementation.

Google's Cloud Vision

Google's Cloud Vision algorithm assigned 6.215 unique labels to the photograph, 3.295 of which occurred less than 10 times, or more than 90.000 times. After removal of these labels, 109 photographs had to be excluded from the dataset, since all the labels assigned to these

photographs occurred less than 10 times throughout the dataset. The dataset that was analysed with a Structural Topic Model therefore contained information from 195.091 photographs. The most frequent label was “water”, which was assigned to 63.600 photographs, followed by “mountain”, “cloud”, and “highland”, which occurred respectively 59.909, 47.186, and 46.319 times (Figure 1).

Figure 1 – Word cloud of all photograph labels. The fond size represents the prevalence of each word in the dataset.

Structural topic model

A structural topic model was used to identify 8 topics within the labels for each picture (Table 2). Despite the overlap between some of these topics, each topic can be distinguished from the other topics based on sensible criteria. For example, topics 1, 2 and 8 all appear to be associated with human activities, in particular: arts & events (topic 1), ships & harbours (topic 2), and houses & towns (topic 8). Topics 3 - 7 are all related to nature, wildlife, and outdoor activities. These topics represent nature-based tourism in 5 different categories: water (topic 3), night & northern lights (topic 4), mountains (topic 5), snow & winter (topic 6), and plants & wildlife (topic 7). Based on the prevalence of keywords within each identified topic, we assigned a single-word description to each topic (Table 2). These descriptions are only a general representation of each topic, which may not appropriately capture each photo that was assigned to the topic. Some labels (e.g. water) occur in several topics. Overall, the most prevalent topic was topic 3 (water), while the prevalence of topics 4 to 8 was relatively equal (Table 2). Topics 1 (events) and 2 (boats) were the least prevalent, but each still contributed to approximately 8% of the data (Table 2).

Table 2 – Overall topic prevalence, and the 7 most prevalence words within each topic

Topic	***One-word description***	***Top 7 keywords***	***Expected proportions***

1	Events	Event, Performance, Music, Entertainment, Performing Arts, Musician, Stage	0.08
2	Boats	Boat, Water, Watercraft, Waterway, Water transportation, Ship, Harbour	0.08
3	Water	Water, Sea, Loch, Lake, Horizon, Coast, Cloud	0.22
4	Night	Atmosphere, Night, Phenomenon, Darkness, Atmosphere of earth, Aurora, Nature	0.13
5	Mountains	Mountain, Highland, Landscape, Fell, Hill, Mountain range, Mountainous landforms	0.14
6	Winter	Snow, Winter, Freezing, Glacial landform, Geological phenomenon, Ice, Mountain range	0.12
7	Flora & Fauna	Grass, Plant, Tree, Wildlife, Bird, Grassland, Ecoregion	0.11
8	Urban	House, Home, Building, Road, Wood, Tree, Town	0.12

Influence of corona measures on photographer preference

We used the STM to study the influence of corona measures in Norway on the prevalence of photography topics. We marked April 1st, 2020 as the initiation of corona measures in Norway. After the implementation of corona measures, international photographers focused more on topics 5 (Mountains), 6 (Winter) and 7 (Flora & Fauna), while topics 1, 2, 3, and 4 (Events, Boats, Water, and Night) became less important (Figure 2). The change in prevalence for topic 7 (Flora & Fauna) was approximately 8 %. Topic 8 (Urban) remained equally prevalent (no statistical difference). Amongst Norwegian photographers, topics 5, 6 and 8 (Mountains, Winter, and Urban) became more prevalent after corona measures were implemented, while topics 1 (Events) and 4 (Night) became less important after the implementation of corona measures. For Norwegian photographers, topics 2 (Boats), 3 (Water), and 7 (Flora & Fauna) remained equally prevalent (Figure 2).

Figure 2 – Topic covariate levels (in proportions) after corona measures, compared to before corona measures. Covariate levels > 0 indicate the topic was more prevalent after corona measures, compared to before the corona measures. The graph on the left illustrates changes in covariate levels after corona measures for international photographers, while the right graph illustrates changes in covariate levels for Norwegian photographers.

Figure 2 illustrates the change in prevalence of each topic for international and domestic photographers separately, but it does not show the importance of topics for one group of photographers compared to the other group (international or domestic). Therefore, we also compared the influence of photographer's nationality on topic prevalence before and after corona (Figure 3). Topic 1 (Events) and topic 4 (Night) were more prevalent amongst Norwegian photographers, compared to international photographers, both before and after corona measures. Topics 3 (Water), 5 (Mountains), and 6 (Winter) were more prevalent amongst international photographers, both before and after corona measures. Topic 7 (Flora & Fauna) became more prevalent amongst international photographers after the corona measures. The opposite is true for topic 2 (Boats), which became more prevalent amongst Norwegian photographers after corona measures. After corona measures, topic 8 (Urban) became more important for Norwegian photographers, compared to international photographers, while there had not been a significant difference before (Figure 3).

Figure 3 – Compared covariate levels for international photographers compared to Norwegians. Covariate levels > 0 indicate that the topic was more popular (prevalent) amongst international photographers, while negative values indicate a prevalence amongst Norwegian photographers. The left graph shows estimated covariate levels before corona measures, while the graph on the right illustrates covariate levels after corona measures.

Discussion

Nature forms the core of recreation and tourism in northern Norway, as reflected in the labels and topics that were assigned to photographs from the area. Amongst the most occurring labels were "water", "mountains" and "clouds", while the most prevalent topic centred around coastal photography. The bulk of the photographers that shared content from northern Norway on the Flickr platform was of international origin, but numbers of both domestic and international photographers decreased sharply after the implementation

of measures to combat the spread of COVID-19. Both international and domestic photographers focused more on the topics “winter” and “mountains” after the implementation of COVID-19 measures, and less on the topics “events” and “night”. Domestic photographers differed from international photographers as the topic “urban” became more prevalent in this group. Before the implantation of COVID-19 measures, international photographers focused more on the topic “boats” than domestic photographers, but this topic became most prevalent amongst domestic photographers during the pandemic. The opposite is true for topic “Flora & Fauna”, which was most prevalent amongst domestic photographers before the pandemic but preferred by international photographers during the pandemic.

The onset of the global COVID-19 pandemic, and the measures to combat it’s spread have severely impacted tourism and recreation around the world (Sigala, 2020). National and international measures hinder international and domestic travels, which leads to a reduction of the number of visitors, particularly in remote areas (Spenceley et al., 2021). In northern Norway, the reduction of international visitors was particularly high, compared to the European or global average (World Tourism Organization United Nations, 2021). This reduction is also reflected in the annual number of photographers to post photographs from northern Norway on the Flickr platform. Although a decline in the number of photographers started in 2015, the rate of decline increased dramatically in 2020 compared to the previous years. The number of photographers in 2020 was 52% less compared to 2019 and reached the lowest level since 2006. Our results show a clear difference between international and domestic visitors, as the number of international visitors decreased by 54% from 2019 to 2020, compared to a 17% reduction amongst domestic visitors. However, it should be noted that in the last decade, domestic visitors form less than a quarter of the total number of

visitors in northern Norway, according to our data. Some of the domestic photographers may have been identified erroneously as international visitors, as the nationality of some photographers had to be estimated from the geographic distribution of their photographs. It is possible that a well-travelled Norwegian photographer is mis-identified as an international photographer. Nonetheless, it appears Norwegian domestic tourists are underrepresented on the Flickr platform.

In addition to reduced numbers of visitors, the COVID-19 pandemic also affected the behaviour of domestic and international visitors. Overall, the topics “events”, “nights” and “urban” are mostly associated with domestic photographers, both before and during the pandemic, while the topics “water”, “mountains” and “winter” are commonly dominated by international photographers (Figure 3). During the pandemic, the prevalence for the topics “mountains” and “winter” increased amongst both domestic and international photographers (Figure 2). Recreation that is associated with these topics requires only limited interaction with tour operators, nature guides, or other visitors, and can be enjoyed by individuals in relative isolation. In contrast, the topic “events” became less prevalent amongst both international and domestic photographers. The latter topic often includes a social component (exhibitions, concerts, performances etc.), which has been restricted during the pandemic. Domestic photographers showed an increased focus on the topic “urban” during the pandemic, while the prevalence did not change amongst international photographers. This could indicate that during the pandemic, domestic photographers engaged in alternative touristic activities, such as visiting remote, private owned cabins to escape from the risk of infection in densely populated areas. Note that this topic does not exclusively refer to urban activities, but also includes the photography of buildings, houses, roads and even towns, which may not be affected by a reduction in social activities.

International visitors did not appear to switch to these alternative touristic activities, since the prevalence of this topic did not change amongst their group. These patterns suggest that under highly restrictive conditions, domestic visitors may be more flexible in finding alternative touristic activities, while international visitors may be relatively rigid in the consumption of nature-based products, especially in remote areas where the availability of alternative (e.g. cultural) attractions are sparse.

International and domestic photographers responded differently to the pandemic. During the pandemic, international photographers took fewer photos within the topics “boats” and “water”, which could be explained by a decreased availability of organised or guided boat trips, due to COVID-19 measures. At the same time, the accessibility to (private-owned) boats may not have been restricted as much for domestic photographers, which could explain why this topic remained equally prevalent during the pandemic. As a result of these changes, the topic “boats” became dominated by domestic photographers during the pandemic, while it was dominated by international photographers before the pandemic (Figure 3). Before the pandemic, the topic “flora & fauna” was dominated by domestic photographers, but during the pandemic it became more important for international photographers. The prevalence of this topic increased dramatically amongst international photographers, while it remained the same amongst domestic photographers. There are a few possible explanations for the increase amongst international photographers. First, similarly to recreation that was associated with the topics “mountains” and “winter”, the topic “flora & fauna” can be enjoyed in relative isolation, as it does not necessarily depend on organised trips. Second, and in connection to this, the availability of recreational options that are associated to other photography topics may have been reduced during the pandemic. For example, options for organised boat trips may have been limited, due to

regulations regarding social distance and maximum group size. Thirdly, the increase in prevalence of the topic “flora & fauna” amongst international photographers may be attributed to an increased interest in nature and wildlife, fuelled by the effects of the pandemic back home. Finally, the international photographers that visited northern Norway during the pandemic may not be representative of the international photographers that would visit northern Norway if there was no pandemic. As it is more difficult to enter Norway during the pandemic, this group of visitors is likely highly motivated to focus on the most meaningful attractions, which happen to be nature oriented for this core group of visitors that are willing to undergo all the travel restrictions, quarantine, and limited availability of organized tours, thus representing the core values of the visitor preferences in a remote area like northern Norway. Our results indicate that under highly restricted conditions, both international and domestic visitors shift towards tourism activities that may not be dependent on tour operators. This means an additional pressure for the tourism industry in a remote area such as northern Norway, besides the pressure of reduced visitor numbers. Alternatively, our results may be caused by a reduction in the offer of organised tourism activities during the pandemic, which would stimulate visitors to find activities that can be done independently. In either case, this study warrants a careful evaluation of the role of the nature-based tourism industry in remote areas, to ensure the sustainability of the industry in the future.

In conclusion, our study indicates that domestic visitors have a high capacity to adapt to restrictive environments under the pandemic scenario and shift from nature-based activities to urban activities. International visitors, on the other hand, show little variation in preferences, indicating that for them, the main touristic product of remote areas such as northern Norway is mainly natural. Both international and domestic visitors appeared to

target tourism activities that can be enjoyed individually. This highlights the urgency of collaborative efforts between local municipalities and tour operators to ensure the long-term sustainability of local nature-based tourism products.

References

Derks, J., Giessen, L., & Winkel, G. (2020). COVID-19-induced visitor boom reveals the importance of forests as critical infrastructure. *Forest Policy and Economics*, *118*, 102253. https://doi.org/10.1016/j.forpol.2020.102253

Ghermandi, A., Camacho-Valdez, V., & Trejo-Espinosa, H. (2020). Social media-based analysis of cultural ecosystem services and heritage tourism in a coastal region of Mexico. *Tourism Management*, *77*, 104002. https://doi.org/10.1016/j.tourman.2019.104002

Grimmer, J., & Stewart, B. M. (2013). Text as data: The promise and pitfalls of automatic content analysis methods for political texts. *Political Analysis*, *21*(3), 267–297. https://doi.org/10.1093/pan/mps028

Innovasjon Norge. (2019). *Key Figures for Norwegian Travel and Tourism* (p. 75). https://assets.simpleviewcms.com/simpleview/image/upload/v1/clients/norway/Key_figures_2019_70ab0c61-9c91-4b2a-b450-63898baceccc.pdf

Jaung, W. (2021). Changes in human-nature relations during pandemic outbreaks: a big data analysis. *Science of the Total Environment*, *768*, 144530. https://doi.org/10.1016/j.scitotenv.2020.144530

Li, J., Xu, L., Tang, L., Wang, S., & Li, L. (2018). Big data in tourism research: A literature review. *Tourism Management*, *68*, 301–323. https://doi.org/10.1016/j.tourman.2018.03.009

Lucas, C., Nielsen, R. A., Roberts, M. E., Stewart, B. M., Storer, A., & Tingley, D. (2015). Computer-assisted text analysis for comparative politics. *Political Analysis*, *23*(2), 254–277. https://doi.org/10.1093/pan/mpu019

Mancini, F., Coghill, G. M., & Lusseau, D. (2018). Using social media to quantify spatial and temporal dynamics of nature-based recreational activities. *PLOS ONE*, *13*(7), e0200565. https://doi.org/10.1371/JOURNAL.PONE.0200565

Morse, J. W., Gladkikh, T. M., Hackenburg, D. M., & Gould, R. K. (2020). COVID-19 and human-nature relationships: Vermonters' activities in nature and associated nonmaterial values during the pandemic. *PLOS ONE*, *15*(12), e0243697. https://doi.org/10.1371/journal.pone.0243697

Muñoz, L., Hausner, V., Brown, G., Runge, C., & Fauchald, P. (2019). Identifying spatial overlap in the values of locals, domestic- and international tourists to protected areas. *Tourism Management*, *71*, 259–271. https://doi.org/10.1016/j.tourman.2018.07.015

Muñoz, L., Hausner, V. H., Runge, C., Brown, G., & Daigle, R. (2020). Using crowdsourced spatial data from Flickr vs. PPGIS for understanding nature's contribution to people in Southern Norway. *People and Nature*, *2*(2), 437–449. https://doi.org/10.1002/pan3.10083

OECD. (2020). Tourism Policy Responses to the coronavirus (COVID-19). *Oecd*, *June*, 1–50. https://read.oecd-ilibrary.org/view/?ref=124_124984-7uf8nm95se&Title=Covid-19: Tourism Policy Responses

Peng, G., Liu, Y., Wang, J., & Gu, J. (2017). Analysis of the prediction capability of web search data based on the HE-TDC method – prediction of the volume of daily tourism visitors.

Journal of Systems Science and Systems Engineering 2017 26:2, *26*(2), 163–182. https://doi.org/10.1007/S11518-016-5311-7

R Core Team. (2020). *R: A language and environment for statistical computing. R Foundation for Statistical Computingitle*. https://www.r-project.org/

Randler, C., Tryjanowski, P., Jokimäki, J., Kaisanlahti-Jokimäki, M.-L., & Staller, N. (2020). SARS-CoV2 (COVID-19) Pandemic Lockdown Influences Nature-Based Recreational Activity: The Case of Birders. *International Journal of Environmental Research and Public Health*, *17*(19), 7310. https://doi.org/10.3390/ijerph17197310

Rice, W. L., & Pan, B. (2021). Understanding changes in park visitation during the COVID-19 pandemic: A spatial application of big data. *Wellbeing, Space and Society*, *2*, 100037. https://doi.org/10.1016/j.wss.2021.100037

Richards, D. R., Tunçer, B., & Tunçer, B. (2018). Using image recognition to automate assessment of cultural ecosystem services from social media photographs. *Ecosystem Services*, *31*, 318–325. https://doi.org/10.1016/j.ecoser.2017.09.004

Roberts, M. E., Stewart, B. M., & Tingley, D. (2019). Stm: An R package for structural topic models. *Journal of Statistical Software*, *91*(1), 1–40. https://doi.org/10.18637/jss.v091.i02

Roberts, M. E., Stewart, B. M., Tingley, D., Lucas, C., Leder-Luis, J., Gadarian, S. K., Albertson, B., & Rand, D. G. (2014). Structural topic models for open-ended survey responses. *American Journal of Political Science*, *58*(4), 1064–1082. https://doi.org/10.1111/ajps.12103

Rose, S., Suri, J., Brooks, M., & Ryan, P. G. (2020). COVID-19 and citizen science: lessons

learned from southern Africa. In *Ostrich* (pp. 188–191). Taylor & Francis. https://doi.org/10.2989/00306525.2020.1783589

Runge, C. A., Hausner, V. H., Daigle, R. M., & Monz, C. A. (2020). Pan-Arctic analysis of cultural ecosystem services using social media and automated content analysis. *Environmental Research Communications*, *2*(7), 075001. https://doi.org/10.1088/2515-7620/ab9c33

Rutz, C., Loretto, M. C., Bates, A. E., Davidson, S. C., Duarte, C. M., Jetz, W., Johnson, M., Kato, A., Kays, R., Mueller, T., Primack, R. B., Ropert-Coudert, Y., Tucker, M. A., Wikelski, M., & Cagnacci, F. (2020). COVID-19 lockdown allows researchers to quantify the effects of human activity on wildlife. In *Nature Ecology and Evolution* (Vol. 4, Issue 9, pp. 1156–1159). Nature Publishing Group. https://doi.org/10.1038/s41559-020-1237-z

Shilling, F., Nguyen, T., Saleh, M., Kyaw, M. K., Tapia, K., Trujillo, G., Bejarano, M., Waetjen, D., Peterson, J., Kalisz, G., Sejour, R., Croston, S., & Ham, E. (2021). A Reprieve from US wildlife mortality on roads during the COVID-19 pandemic. *Biological Conservation*, *256*, 109013. https://doi.org/10.1016/j.biocon.2021.109013

Shoval, N., & Ahas, R. (2016). The use of tracking technologies in tourism research: the first decade. *Tourism Geographies*, *18*(5), 587–606. https://doi.org/10.1080/14616688.2016.1214977

Sigala, M. (2020). Tourism and COVID-19: Impacts and implications for advancing and resetting industry and research. *Journal of Business Research*, *117*, 312–321. https://doi.org/10.1016/j.jbusres.2020.06.015

Soga, M., Evans, M. J., Cox, D. T. C., & Gaston, K. J. (2021). Impacts of the COVID-19

pandemic on human–nature interactions: Pathways, evidence and implications. *People and Nature*, *3*(3), 518–527. https://doi.org/10.1002/pan3.10201

Spalding, M., Burke, L., & Fyall, A. (2020). Covid-19: implications for nature and tourism. *Anatolia*, *32*(1), 1–2. https://doi.org/10.1080/13032917.2020.1791524

Spenceley, A., McCool, S., Newsome, D., Báez, A., Barborak, J. R., Blye, C. J., Bricker, K., Cahyadi, H. S., Corrigan, K., Halpenny, E., Hvenegaard, G., King, D. M., Leung, Y. F., Mandić, A., Naidoo, R., Rüede, D., Sano, J., Sarhan, M., Santamaria, V., … Zschiegner, A. K. (2021). Tourism in protected and conserved areas amid the covid-19 pandemic. *Parks*, *27*(Special Issue), 103–118. https://doi.org/10.2305/IUCN.CH.2021.PARKS-27-SIAS.en

Teschner, F. (2021). *RoogleVision: to Google's Cloud Vision API for Image Recognition, OCR and Labeling.* (0.0.1.1.).

Toivonen, T., Heikinheimo, V., Fink, C., Hausmann, A., Hiippala, T., Järv, O., Tenkanen, H., & Di Minin, E. (2019). Social media data for conservation science: A methodological overview. In *Biological Conservation* (Vol. 233, pp. 298–315). Elsevier Ltd. https://doi.org/10.1016/j.biocon.2019.01.023

Venter, Z. S., Barton, D. N., Gundersen, V., Figari, H., & Nowell, M. (2020). Urban nature in a time of crisis: Recreational use of green space increases during the COVID-19 outbreak in Oslo, Norway. *Environmental Research Letters*, *15*(10), 104075. https://doi.org/10.1088/1748-9326/abb396

Wood, S. A., Winder, S. G., Lia, E. H., White, E. M., Crowley, C. S. L., & Milnor, A. A. (2020). Next-generation visitation models using social media to estimate recreation on public

lands. *Scientific Reports*, *10*(1), 1–12. https://doi.org/10.1038/s41598-020-70829-x

World Tourism Organization United Nations. (2021). *International Tourism and covid-19 UNWTO*. Unwto Global Tourism Dashboard. https://www.unwto.org/international-tourism-and-covid-19

Figure Click here to access/download;Figure;FIGURES.docx

Figures for: "Impact of the COVID-19 pandemic on human-nature relations in a remote nature-based tourism destination"

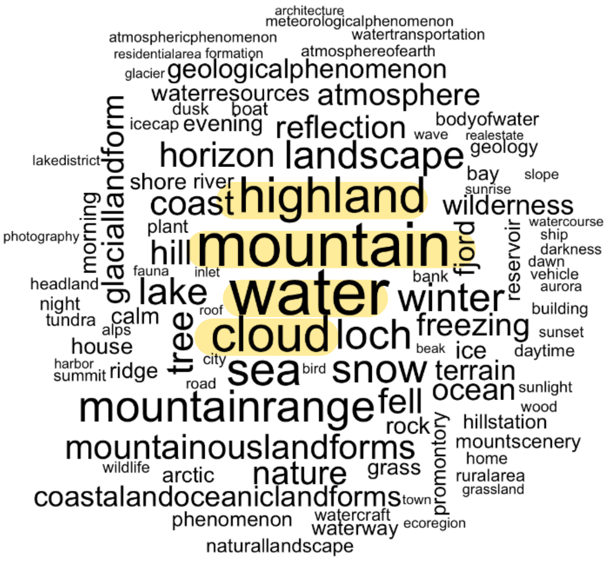

Figure 1 – Word cloud of all photograph labels. The fond size represents the prevalence of each word in the dataset.

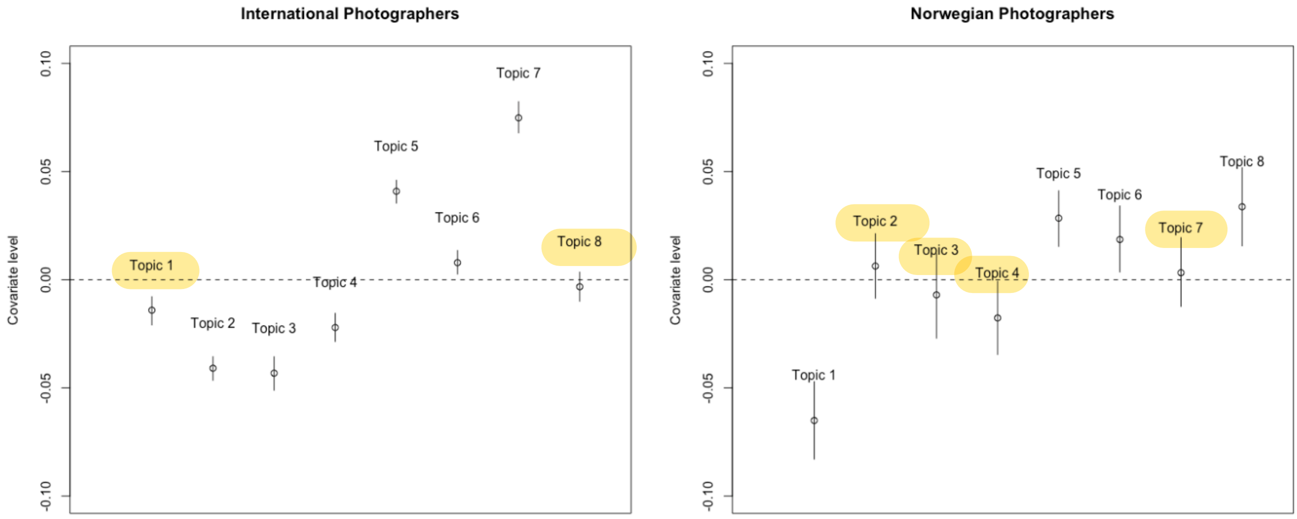

Figure 2 – Topic covariate levels (in proportions) after corona measures, compared to before corona measures. Covariate levels > 0 indicate the topic was more prevalent after corona measures, compared to before the corona measures. The graph on the left illustrates changes in covariate levels after corona measures for international photographers, while the right graph illustrates changes in covariate levels for Norwegian photographers.

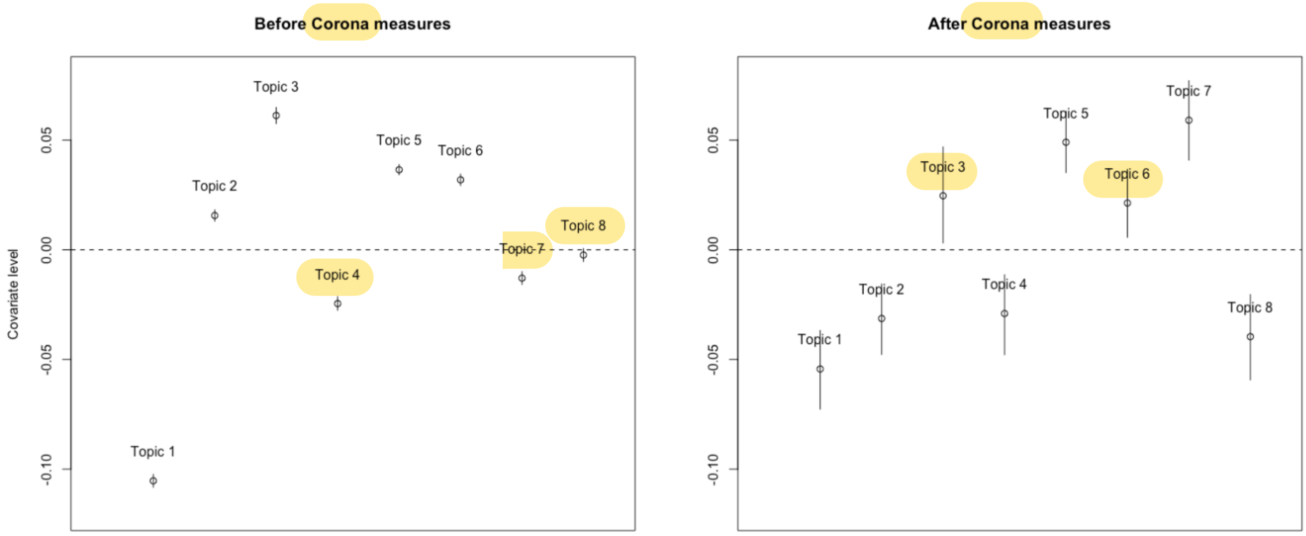

Figure 3 – Compared covariate levels for international photographers compared to Norwegians. Covariate levels > 0 indicate that the topic was more popular (prevalent) amongst international photographers, while negative values indicate a prevalence amongst Norwegian photographers. The left graph shows estimated covariate levels before corona measures, while the graph on the right illustrates covariate levels after corona measures.
